# Measurement error of mean sac diameter and crown-rump length among pregnant women at Mulago hospital, Uganda

**DOI:** 10.1186/s12884-018-1769-2

**Published:** 2018-05-04

**Authors:** Sam Ali, Rosemary Kusaba Byanyima, Sam Ononge, Jerry Ictho, Jean Nyamwiza, Emmanuel Lako Ernesto Loro, John Mukisa, Angella Musewa, Annet Nalutaaya, Ronald Ssenyonga, Ismael Kawooya, Benjamin Temper, Achilles Katamba, Joan Kalyango, Charles Karamagi

**Affiliations:** 1 Clinical Epidemiology Unit, Makerere University College of Health Sciences, P.O. Box 7072, Kampala, Uganda; 2 Department of Radiology, UMC Victoria Hospital Bukoto, P.O. Box 72587, Kampala, Uganda; 3 Department of Radiology, Mulago Hospital Complex, P.O. Box 7051, Kampala, Uganda; 4 Department of Obstetrics and Gynaecology, Makerere University College of Health Sciences, P.O. Box 7072, Kampala, Uganda; 5 Department of Medicine, Makerere University College of Health Sciences, P.O. Box 7072, Kampala, Uganda; 6 Department of Pharmacy, Makerere University College of Health Sciences, P.O. Box 7072, Kampala, Uganda; 7 Department of Pediatrics and Child Health, Makerere University College of Health Sciences, P.O. Box 7072, Kampala, Uganda

**Keywords:** Mean sac diameter, Crown-rump length, Measurement error

## Abstract

**Background:**

Ultrasonography is essential in the prenatal diagnosis and care for the pregnant mothers. However, the measurements obtained often contain a small percentage of unavoidable error that may have serious clinical implications if substantial. We therefore evaluated the level of intra and inter-observer error in measuring mean sac diameter (MSD) and crown-rump length (CRL) in women between 6 and 10 weeks’ gestation at Mulago hospital.

**Methods:**

This was a cross-sectional study conducted from January to March 2016. We enrolled 56 women with an intrauterine single viable embryo. The women were scanned using a transvaginal (TVS) technique by two observers who were blinded of each other’s measurements. Each observer measured the CRL twice and the MSD once for each woman. Intra-class correlation coefficients (ICCs), 95% limits of agreement (LOA) and technical error of measurement (TEM) were used for analysis.

**Results:**

Intra-observer ICCs for CRL measurements were 0.995 and 0.993 while inter-observer ICCs were 0.988 for CRL and 0.955 for MSD measurements. Intra-observer 95% LOA for CRL were ± 2.04 mm and ± 1.66 mm. Inter-observer LOA were ± 2.35 mm for CRL and ± 4.87 mm for MSD. The intra-observer relative TEM for CRL were 4.62% and 3.70% whereas inter-observer relative TEM were 5.88% and 5.93% for CRL and MSD respectively.

**Conclusions:**

Intra- and inter-observer error of CRL and MSD measurements among pregnant women at Mulago hospital were acceptable. This implies that at Mulago hospital, the error in pregnancy dating is within acceptable margins of ±3 days in first trimester, and the CRL and MSD cut offs of ≥7 mm and ≥ 25 mm respectively are fit for diagnosis of miscarriage on TVS. These findings should be extrapolated to the whole country with caution. Sonographers can achieve acceptable and comparable diagnostic accuracy levels of MSD and CLR measurements with proper training and adherence to practice guidelines.

## Background

The advent of ultrasonography and its swift advances has in the recent years significantly improved prenatal diagnosis and care globally [1, 2]. In the early stages of a pregnancy, ultrasound is essential in predicting the risk of adverse pregnancy outcomes such as aneuploidy, stillbirth, pre-eclampsia and the possibility of abnormal cord insertion visualization [3, 4]. It is also used for fetal anatomic surveys during a second-trimester scan to detect fetal malformations, monitoring fetal growth in utero and in pregnancy dating [5–7]. Therefore, given the essential role of ultrasonography in clinical decision making, it is imperative that sonographic parameters obtained are accurate and precise [8]. However, a small percentage of error in measurements or incompleteness of the information obtained is at times unavoidable. [9, 10]. In first trimester, measurement error of CRL and MSD has been reported to be ±18.78% limits of agreement in United Kingdom (UK) [11]. If significant, this error has implications on the accuracy of estimates of the fetal gestation age obtained. And if not taken into account at MSD or CRL cut offs used for the diagnosis of miscarriage, some normal pregnancies may be erroneously deemed non-viable [11]. Consequently, this could lead to inadvertent termination of viable embryos and immense physical and emotional harm to the patient [11–13].

The unavoidable measurement error or incompleteness in information obtained during an ultrasound examination is related to various factors including but not limited to the skill of the sonographer and their level of training; technical factors related to the patient such as body habitus; the quality of the machine; fetal position; and the duration of the examination [14]. As in other low resourced settings, Uganda’s healthcare system faces severe shortage of imaging experts [15–17]. This results in high workload which affects the performance and efficiency of health workers. In addition, majority of the low-income countries lack adequate resources to acquire high-end ultrasound machines with very good spatial resolution [16, 18]. With low spatial resolution machines, images appear blurred or enlarged, and due to this effect, calipers are placed beyond or may not cover the true dimensions leading to errors in measurements [19]. Errors arising from variation between machines have been found to be substantial [19]. The Ministry of Health Standards on Diagnostic Imaging and Therapeutic Radiology in Uganda recommends the use of CRL cut off of 5 mm to diagnose a miscarriage yet this has changed following recommendation by recent studies. The use of the outdated CRL cut off of 5 mm increases the risk of misdiagnosing normal pregnancies. This practice guidelines does not also provide clear guidance for measurement of MSD [20]. This may lead to significant variations in MSD measurements.

The reliability of CRL and MSD measurements in first trimester using modern ultrasound equipment has not been adequately explored in the low developed countries like in the developed nations [11, 19, 21]. This study sought to understand the level of intra- and inter-observer variability in measuring MSD and CRL in women between 6 and 10 weeks’ gestation at Mulago National Referral Hospital.

## Methods

This was a cross-sectional study conducted on pregnant women at the Department of Obstetrics and Gynecology, Mulago National Referral Hospital, Uganda from January to March 2016. We consecutively enrolled women with a single viable intrauterine embryo from 6 to 10 weeks of gestation and not bleeding. The first observer examined a woman who had consented, to assess if they were eligible for inclusion in this study. The second observer then further examined the eligible participant. The two observers examined each woman at the same point in time. Both observers used a Phillips Envisor (PHILIPS, USA, 2009) with a 7.5 MHz transvaginal probe for B-imaging to do all examinations.

**Fig. 1:**
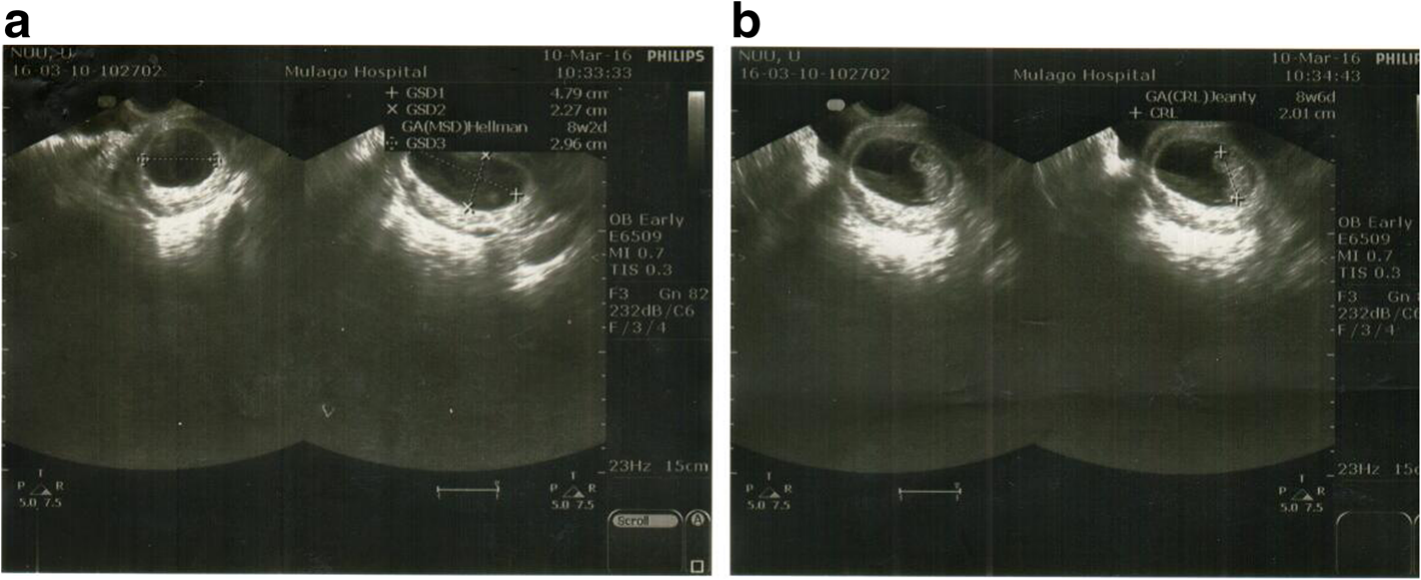
**a** Measurement of mean sac diameter at 8 weeks’ gestational age using transvaginal ultrasound scan. Gestational sac diameter was obtained by placing the calipers inner-to-inner on the sac wall, excluding the surrounding echogenic rim of tissue. MSD was calculated by first adding the longitudinal, anteroposterior and transverse dimensions of the chorionic cavity. Thereafter, the sum of the three measurements was divided by three. **b** Measurement of crown–rump length with transvaginal ultrasound at 8 weeks’ gestational age. CRL was measured as the maximal straight-line length of the embryo, obtained along its longitudinal axis, with the embryo neither too flexed nor too extended

For each examined participant, the observers took CRL measurements twice and MSD measurements once, and in between the two CRL measurements, the observers examined the ovaries and uterus. These measurements were obtained as described in the WHO Manual of diagnostic ultrasound, Volume 2 [5] (Fig. 1). To archive blinding, the measurements of the first observer were removed from the machine before the second observer was allowed to enter the examination room. The same two sonographers that examined all the women had good training in obstetric sonography and at least five years of experience in fetal ultrasound. A female nurse or professional was always brought into the examination room for all the transvaginal ultrasound scans done by the male sonographer to make the women feel comfortable and safe.

### Statistical issues

#### Sample size

The sample size calculations were based on the formula below by considering 95% Limits of agreement (LOA) of ±18.78% as the cut off for clinical significance [11, 22, 23]. In the formula, n = desired sample size and s = standard deviation of the differences in CRL or MSD measurements [24].

$$ 1.96\kern0.5em \sqrt{\left[\frac{3{\mathrm{s}}^2}{\mathrm{n}}\right]}\kern0.5em =\kern0.5em \mathrm{Desired}\ \mathrm{confidence}\ \mathrm{interval}\ \mathrm{of}\ \mathrm{limits}\ \mathrm{of}\ \mathrm{agreement}\ \left[24\right]. $$

#### Statistical analysis

Data was double entered and validated in Epidata version 3.1 to identify inconsistent entries before being exported to SPSS Version 19.0 for analysis. Scatterplots of paired sets of measurements created with the line of equality were visually assessed for potential systematic errors in the intra and inter-observer measurements. A paired t-test at 0.05 set level of significance was used to check if the paired sets of measurements were significantly different, to rule out any systematic errors in the measurements.

To assess the strength of the absolute agreement within and between observers, the intraclass correlation coefficient (ICC) was computed based on a two-way random effects model [24–26]. Normality, constant mean and variance assumptions for LOA were fulfilled. Therefore, the difference between paired sets of measurements were plotted against their mean in Bland–Altman plots to assess the level of clinical agreement within and between the observers. The lack of agreement between measurements or observers becomes relevant only when the LOAs are wider than what is clinically acceptable [27, 28]. Technical error of measurements (TEM) within and between observers were calculated by taking the square root of the sum of the squares of the differences of the paired sets of measurements divided by twice the total number of participants measured.

## Results

We screened 71 pregnant women suspected to be in first trimester and enrolled 56 in this study. Of the 15 women excluded from the study, one had a ruptured ectopic pregnancy; three had empty gestation sacs; six were more than 10 weeks of gestation pregnant; three were not pregnant and two declined to be examined after consenting. The mean (SD) maternal age was 25.8 (4.33) and mean (SD) gestation age was 7.5 (1.14) (Table 1).

**Table 1:** Demographic characteristics of women between 6 and 10 weeks of gestation in Mulago Hospital, Kampala, 2016

Variable	Frequency (*N* = 56)	Percentage (%)
Age		
Mean(SD*)	25.8 (4.33)	
Gravidity		
Median (IQR*)	3 (1.5,4)	
Parity		
Median (IQR*)	1 (0,2)	
Number of previous abortions		
Median (IQR*)	0 (0,1)	
Weight		
Median (IQR*)	54 (50.5,61.0)	
Height		
Median (IQR*)	156.3 (154.0,160.1)	
Gestation age		
Mean (SD*)	7.5 (1.14)	
Body Mass Index		
Underweight (< 18.5)	5	8.9
Normal (18.5–24.9)	39	69.7
Overweight (25.0–29.9)	7	12.5
Obesity (≥ 30.0)	5	8.9

*SD standard deviation, *IQR interquartile range

**Fig. 2:**
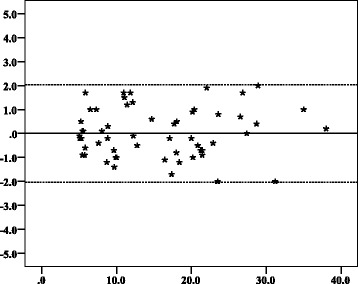
Bland–Altman plots with 95% limits of agreement showing intra-observer agreement of crown–rump length measurements of observer 1. Y axis title: Difference in CRL (mm). Y axis scale = 1. From − 5, − 4, − 3, − 2, − 1, 0, 1, 2, 3, 4, to 5. X axis: Mean of first and second CRL measurements of observer 1 (mm). X axis scale = 10. Start and end point: 0, 10, 20, 30, 40. ▬▬▬▬▬▬ Reference point where the mean difference between repeated measures is equal to zero. ▬ ▬ ▬ ▬ ▬ ▬ The upper and lower limit of the 95% confidence interval of limits of agreement

**Fig. 3:**
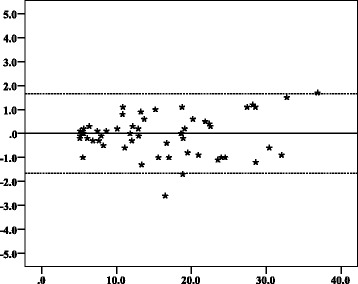
Bland–Altman plots with 95% limits of agreement showing intra-observer agreement of crown–rump length measurements of observer 2. Y axis: Difference in CRL (mm). Y axis scale = 1. From − 5, − 4, − 3, − 2, − 1, 0, 1, 2, 3, 4, to 5. X axis: Mean of first and second CRL measurements of observer 2 (mm). X axis scale = 10. Start and end point: 0, 10, 20, 30, 40. ▬▬▬▬▬▬ Reference point where the mean difference between repeated measures is equal to zero. ▬ ▬ ▬ ▬ ▬ ▬ The upper and lower limit of the 95% confidence interval of limits of agreement

**Fig. 4:**
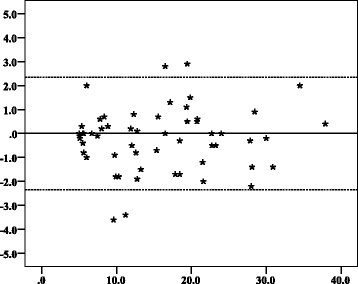
Bland–Altman plots with 95% limits of agreement showing inter-observer agreement of crown–rump length measurements of observer 1 and observer 2. Y axis: Difference in CRL (mm). Y axis scale = 1. From − 5, − 4, − 3, − 2, − 1, 0, 1, 2, 3, 4, to 5. X axis: Mean of CRL measurements of observers 1 and 2 (mm). X axis scale = 10. Start and end point: 0, 10, 20, 30, 40. ▬▬▬▬▬▬ Reference point where the mean difference between repeated measures is equal to zero. ▬ ▬ ▬ ▬ ▬ ▬ The upper and lower limit of the 95% confidence interval of limits of agreement

**Fig. 5:**
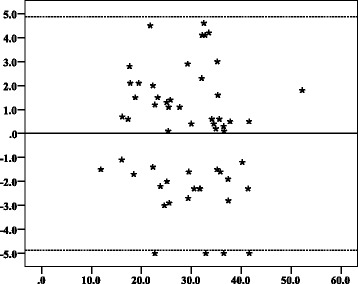
Bland–Altman plots with 95% limits of agreement showing inter-observer agreement of mean gestational sac diameter measurements of observer 1 and observer 2. Y axis title: Difference in MSD (mm). Y axis scale = 1. From − 5, − 4, − 3, − 2, − 1, 0, 1, 2, 3, 4, to 5. X axis title: Mean of MSD measurements of observers 1 and 2 (mm). X axis scale = 10. Start and end point: 0, 10, 20, 30, 40, 50, 60. ▬▬▬▬▬▬ Reference point where the mean difference between repeated measures is equal to zero. ▬ ▬ ▬ ▬ ▬ ▬ The upper and lower limit of the 95% confidence interval of limits of agreement

Intra-observer ICCs were 0.993 and 0.995 for CRL measurements while inter-observer ICCs were 0.988 for CRL and 0.955 for MSD measurements (Table 2). Intra-observer 95% LOAs for CRL were ± 2.04 mm (Fig. 2) and ± 1.66 mm (Fig. 3). Inter-observer 95% LOAs were ± 2.35 mm (Fig. 4) for CRL and ± 4.87 mm for MSD (Fig. 5). Intra-observer relative TEM for CRL were 4.62% and 3.70%, while inter-observer relative TEM were 5.88% for CRL and 5.93% for MSD measurements respectively (Table 3).

**Table 2:** The intraclass correlation coefficients of CRL and MSD measurements of women between 6 and 10 weeks of gestation in Mulago Hospital, Kampala, 2016

Paired set of measurements	ICC*	95% CI*
Intra-observer variation (CRL*)		
Observer 1	0.993	(0.988, 0.996)
Observer 2	0.995	(0.992, 0.997)
Inter-observer variation		
CRL*	0.988	(0.980, 0.993)
MSD*	0.955	(0.924, 0.973)

*CI confidence interval, *ICC Intraclass correlation coefficient, *CRL Crown-rump length, *MSD Mean sac diameter

**Table 3:** The technical error of measurements of CRL and MSD of women between 6 and 10 weeks of gestation in Mulago Hospital, Kampala, 2016

Paired set of measurements	Absolute TEM*	VAV*	Relative TEM* (%)	Classification
Intra-observer variation (CRL*)				
Observer 1	0.72	15.54	4.62	Acceptable
Observer 2	0.58	15.81	3.70	Acceptable
Inter-observer variation				
CRL*	0.92	15.66	5.88	Acceptable
MSD*	1.74	29.36	5.93	Acceptable

*TEM Technical error of measurement, *VAV Variable average value, *CRL Crown-rump length, *MSD Mean sac diameter

## Discussion

This study found a strong observer agreement with intra- and inter-observer ICCs ≥0.955 and this is similar to findings from other studies [29, 30]. Inter-observer 95% limits of agreement for MSD and CRL measurements were also in tandem with findings from other studies [11]. However, intra-observer 95% limits of agreements for CRL measurements were about 2% higher than findings reported in a study by Pexters and colleagues [11]. They reported intra-observer limits of agreement of CRL of ±8.91 and ± 11.37% [11]. The minor differences observed could be attributed to the differences in settings such as observers, patient overload and the finite consistency and read-out precision of the instrument used to measure the structures [9]. The study by Pexters et al. used an ultrasound machine with a 6–12-MHz transvaginal transducer for B-mode imaging while our machine was equipped with a 7.5-MHz probe [11]. Intra-observer inconsistencies highlight a lack of clear or uniform criteria of measurement and interpretation of embryonic landmarks [31]. Detailed instructions in locating landmarks are necessary to minimize intra- and inter-observer technique difference [31]. The majority of our study participants were between 6 to 7 weeks of gestation. At this stage, reproducibility of CRL measurements is better than it is later in the first trimester because of increased embryonic mobility at about 8 weeks’ gestation and above [7]. This could also explain the optimal reliability observed in this study. The relative TEM observed were within clinically acceptable variability in the precision of anthropometric measurements of 5.0% and 7.5% for intra-observer and inter-observer variability respectively [10].

The strength in this study is that it utilized an ultrasound machine with a high spatial resolution. We used the best available ultrasound machine in our setting at the time this study was conducted. This allowed a clear delineation of the anatomical landmarks of the embryo and the gestational sac therefore minimizing measurement errors. In using the same machine, we also eliminated errors due to differences in the machines. The short time interval between intra-observer measurements was our major limitation.

The intra- and inter-observer differences in crown-rump length and mean sac diameter relates to the utility of these measurements in first trimester to accurately estimate gestation age and/or make a diagnosis of early pregnancy loss [5]. If the error is substantial, it may have serious clinical consequences. Our study has shown that intra and inter-observer error of CRL and MSD measurements among pregnant women in our setting were within acceptable limits. Therefore, in relation to the accurate estimation of the gestation age, it is unlikely to result in large differences in days when dating a pregnancy. However, in relation to making a diagnosis of early miscarriage, even a difference of 1 mm can have an impact on the clinical decision [11]. Since our findings are within acceptable limits reported by Pexters et al. and other studies, an MSD cutoff of 25 mm and CRL cutoff of 7 mm for the diagnosis of early miscarriage should be suitable for use in our setting. These cut offs take into account measurement error and were amended as new guidelines [22, 23]. A large multicenter prospective study has demonstrated that these cutoffs are appropriate, with mean gestational sac diameter ≥ 25 mm with an empty sac (364/364 specificity: 100%, 95% confidence interval 99.0% to 100%), embryo with crown-rump length ≥ 7 mm without visible embryo heart activity (110/110 specificity: 100%, 96.7% to 100%) [32].

## Conclusions

Intra- and inter-observer error of CRL and MSD measurements among pregnant women at Mulago hospital were within acceptable limits. This provides assurance that the error in the estimates of gestational age obtained are within acceptable margins of ±3 days in first trimester. The CRL and MSD cut offs of ≥7 mm and ≥ 25 mm are therefore reliable for diagnosis of miscarriage on TVS in our setting. However, these results should be generalized to the rest of the country with caution. Such diagnostic accuracy levels are achievable in Mulago hospital because it is a national referral hospital with sophisticated equipment and highly trained personnel. We recommend further studies in the lower health facilities to establish their diagnostic accuracy levels. Sonographers can achieve acceptable and comparable diagnostic accuracy levels of MSD and CLR measurements with proper training, regular audits and adherence to practice guidelines.

## Abbreviations

CRL: Crown-rump length
ICC: Intra-class correlation coefficient
LOA: Limits of agreement
MSD: Mean sac diameter
TEM: Technical error of measurements

## References

[1] McNay MB, Fleming JE: Forty years of obstetric ultrasound 1957–1997: from A-scope to three dimensions. Ultrasound Med Bio 1999, 25(1):3–56. 10.1016/s0301-5629(98)00129-x 10048801

[2] Alan B, Goya C, Tunc S, Teke M, Hattapoglu S (2016). Assessment of placental stiffness using acoustic radiation force impulse Elastography in pregnant women with fetal anomalies. Korean J Radiol.

[3] Padula F, Laganà A, Vitale S, Mangiafico L, D’Emidio L, Cignini P, Giorlandino M, Gulino F, Capriglione S, Giorlandino C (2016). Ultrasonographic evaluation of placental cord insertion at different gestational ages in low-risk singleton pregnancies: a predictive algorithm. Facts Views Vis ObGyn.

[4] Andrietti S, Carlucci S, Wright A, Wright D, Nicolaides KH. Repeat measurements of uterine artery pulsatility index, mean arterial pressure and serum placental growth factor at 12, 22 and 32 weeks in prediction of pre-eclampsia. Ultrasound Obstet Gynecol. 2017;50(2):221–7. 10.1002/uog.17403 28078815

[5] WHO (2013). WHO manual of diagnostic ultrasound.

[6] Rumack CM: Diagnostic ultrasound, vol. Vol. 1: Elsevier/Mosby; 2011.

[7] Chudleigh Trish, Thilaganathan B: obstetric ultrasound: how, why and when, third edn: Churchill Livingstone; 2004.

[8] Padula F, Capriglione S, Magliarditi M, De Sole R, Nuara R, Santonocito VC, Teodoro MC, Giorlandino C (2015). Goal-directed junior ultrasound training in quantitative measurement of crown-rump length and fetal nuchal translucency: evaluation of a specific training program in a specialized center for prenatal diagnosis. Eur J Obstet Gynecol Reprod Biol.

[9] Harris EF, Smith RN (2009). Accounting for measurement error: a critical but often overlooked process. Arch Oral Biol.

[10] Perini TA, Oliveira GL, Ornellas JD, Oliveira FP (2005). Technical error of measurement in anthropometry. Rev Bras Med Esporte.

[11] Pexsters A, Luts J, Van Schoubroeck D, Bottomley C, Van Calster B, Van Huffel S, Abdallah Y, D'Hooghe T, Lees C, Timmerman D (2011). Clinical implications of intra- and interobserver reproducibility of transvaginal sonographic measurement of gestational sac and crown-rump length at 6-9 weeks' gestation. Ultrasound Obstet Gynecol.

[12] Bickhaus J, Perry E, Schust DJ (2013). Re-examining sonographic cut-off values for diagnosing early pregnancy loss. Gynecol Obstet (Sunnyvale, Calif).

[13] Lubinga SJ, Levine GA, Jenny AM, Ngonzi J, Mukasa-Kivunike P, Stergachis A, Babigumira JB (2013). Health-related quality of life and social support among women treated for abortion complications in western Uganda. Health Qual Life Outcomes.

[14] Padula F, Gulino FA, Capriglione S, Giorlandino M, Cignini P, Mastrandrea ML, D'Emidio L, Giorlandino C (2015). What is the rate of incomplete fetal anatomic surveys during a second-trimester scan?. J Ultrasound Med.

[15] MoH: Health sector development plan 2015-16_2019–20. In. Ministry of Health, Uganda; 2015.

[16] WHO: Medical devices: managing the mismatch: an outcome of the priority medical devices project: World Health Organization; 2010.

[17] Kawooya MG (2012). Training for rural radiology and imaging in sub-saharan Africa: addressing the mismatch between services and population. J Clin Imaging Sci.

[18] Maru DS-R, Schwarz R, Andrews J, Basu S, Sharma A, Moore C (2010). Turning a blind eye: the mobilization of radiology services in resource-poor regions. Glob Health.

[19] Sarris I, Ioannou C, Chamberlain P, Ohuma E, Roseman F, Hoch L, Altman DG, Papageorghiou AT (2012). Intra- and interobserver variability in fetal ultrasound measurements. Ultrasound Obstet Gynecol.

[20] MoH: Standards on Diagnostic Imaging and Therapeutic Radiology in Uganda In.: Ministry of Health 2012: 146.

[21] Souka AP, Pilalis A, Papastefanou I, Salamalekis G, Kassanos D (2012). Reproducibility study of crown-rump length and biparietal diameter measurements in the first trimester. Prenat Diagn.

[22] Lane BF, Wong-You-Cheong JJ, Javitt MC, Glanc P, Brown DL, Dubinsky T, Harisinghani MG, Harris RD, Khati NJ, Mitchell DG (2013). ACR appropriateness criteria® first trimester bleeding. Ultrasound Quarterly.

[23] RCOG: Clinical practice guideline; management of early pregnancy miscarriage. In: Royal College of Obstetricians and Gynaecologists. Edited by Farah Nadine, Nadine Andrea Nugent, Anglim M; 2014.

[24] McAlinden C, Khadka J, Pesudovs K (2011). Statistical methods for conducting agreement (comparison of clinical tests) and precision (repeatability or reproducibility) studies in optometry and ophthalmology. Ophthalmic Physiol Opt.

[25] Weir JP: Quantifying test-retest reliability using the intraclass correlation coefficient and the SEM. J Strength Cond Res 2005, 19(1):231–240. 10.1519/15184.1 15705040

[26] Shrout PE, Fleiss JL (1979). Intraclass correlations: uses in assessing rater reliability. Psychol Bull.

[27] Bland JM, Altman DG (2003). Applying the right statistics: analyses of measurement studies. Ultrasound Obstet Gynecol.

[28] Bland JM, Altman D (1986). Statistical methods for assessing agreement between two methods of clinical measurement. Lancet.

[29] Verburg BO, Mulder PG, Hofman A, Jaddoe VW, Witteman JC, Steegers EA (2008). Intra- and interobserver reproducibility study of early fetal growth parameters. Prenat Diagn.

[30] Verwoerd-Dikkeboom CM, Koning AH, Hop WC, Rousian M, Van Der Spek PJ, Exalto N, Steegers EA (2008). Reliability of three-dimensional sonographic measurements in early pregnancy using virtual reality. Ultrasound Obstet Gynecol.

[31] Kouchi M, Mochimaru M, Tsuzuki K, Yokoi T (1999). Interobserver errors in anthropometry. J Hum Ergol.

[32] Preisler J, Kopeika J, Ismail L, Vathanan V, Farren J, Abdallah Y, Battacharjee P, Van Holsbeke C, Bottomley C, Gould D (2015). Defining safe criteria to diagnose miscarriage: prospective observational multicentre study. BMJ.

